# Primary hyperoxaluria type 2 presenting with febuxostat-associated acute tubular injury in a kidney transplant recipient: a case report

**DOI:** 10.3389/fimmu.2026.1932592

**Published:** 2026-10-28

**Authors:** Qi Qiao, Yun Lu, Chengbiao Xue, Shaojun Ye

**Affiliations:** 1 Department of Pharmacy, Zhongnan Hospital of Wuhan University, Wuhan, China; 2 Zhongnan Hospital of Wuhan University, Institute of Hepatobiliary Diseases of Wuhan University, Transplant Center of Wuhan University, National Quality Control Center for Donated Organ Procurement, Hubei Key Laboratory of Medical Technology on Transplantation, Hubei Clinical Research Center for Natural Polymer Biological Liver, Hubei Engineering Center of Natural Polymer-based Medical Materials, Wuhan, Hubei, China

**Keywords:** acute tubular injury, crystalline nephropathy, GRHPR gene, primary hyperoxaluria type 2, renal allograft

## Abstract

**Background:**

Febuxostat, a non-purine selective xanthine oxidase inhibitor, is increasingly used for managing hyperuricemia in kidney transplant recipients. While generally considered safe in this population, emerging post-marketing data raise concerns about potential nephrotoxicity. Primary hyperoxaluria type 2 (PH2) is a rare autosomal recessive disorder caused by mutations in the GRHPR gene, leading to oxalate overproduction, recurrent nephrolithiasis, and progressive renal failure. Isolated kidney transplantation in PH2 patients carries a high risk of recurrent oxalate nephropathy, which can lead to rapid allograft failure. However, PH2 is often diagnosed late or missed entirely.

**Case summary:**

A 67-year-old woman with end-stage renal disease secondary to bilateral nephrolithiasis underwent deceased-donor kidney transplantation. Post-transplant recovery was initially uneventful, with serum creatinine declining to a nadir of 116.4 μmol/L. She developed hyperuricemia and was started on febuxostat 40 mg daily approximately two months after transplantation. About five months after febuxostat initiation, she presented with progressive, unexplained creatinine elevation from baseline to a peak of 327.9 μmol/L (~ 2.8 times the baseline). Comprehensive diagnostic work-up excluded T cell-mediated rejection, antibody-mediated rejection, BK virus nephropathy, and calcineurin inhibitor toxicity. The first renal allograft biopsy showed acute tubular injury with crystalline material suggestive of drug or phosphate/oxalate deposition; the second biopsy suggested extensive calcium oxalate crystal deposition on polarized light microscopy. Von Kossa staining was positive in both biopsies, implicating calcium phosphate deposition and supporting a mixed calcium oxalate/calcium phosphate crystal composition. Febuxostat was discontinued, and renal function stabilized temporarily but subsequently declined. Metabolic evaluation revealed markedly elevated urinary oxalate excretion. Genetic testing identified a homozygous likely pathogenic splice-site variant c.599-1G>C in the GRHPR gene, confirming the diagnosis of PH2.

**Conclusion:**

This case highlights that PH2 can present as recurrent oxalate nephropathy after kidney transplantation. Febuxostat may have contributed to the clinical presentation, but its causal role remains unproven. Clinicians should consider PH in kidney transplant recipients with unexplained allograft dysfunction and calcium oxalate crystal deposition on biopsy, particularly those with a history of nephrolithiasis of unclear etiology. Early recognition and genetic confirmation are essential to guide appropriate management, including metabolic therapy and consideration of combined liver-kidney transplantation.

## 1 Introduction

Post-transplant hyperuricemia is a common metabolic complication in kidney transplant recipients, affecting approximately 40–60% of patients (1). Febuxostat, a non-purine selective xanthine oxidase inhibitor, has emerged as an alternative to allopurinol for patients with intolerance or inadequate response (2). Multiple meta-analyses and cohort studies have evaluated febuxostat safety in kidney transplant recipients and concluded an acceptable safety profile based on preserved estimated glomerular filtration rate (eGFR) and stable immunosuppressant levels (2, 3). However, post-marketing surveillance data have identified acute renal failure as an adverse event associated with febuxostat, with a reporting odds ratio of 5.67 (95% confidence interval, 5.05–6.36) compared with other drugs (4). Reported cases include acute interstitial nephritis and crystalline nephropathy (5).

Primary hyperoxaluria type 2 (PH2) is a rare autosomal recessive inborn error of metabolism caused by mutations in the glyoxylate reductase/hydroxypyruvate reductase (GRHPR) gene, which encodes the GRHPR enzyme (6). Deficiency of this enzyme induces the systemic overproduction of oxalate, leading to recurrent nephrolithiasis, nephrocalcinosis, and progressive kidney failure (7). PH2 accounts for approximately 10% of all PH cases and has an estimated prevalence of less than 1/1,000,000 (8, 9). PH2 is often late or misdiagnosed because of its rarity and heterogeneous presentation (10). Patients may present with end-stage renal disease of unclear etiology, and the diagnosis is frequently established only after kidney transplantation, when recurrent oxalate nephropathy develops in the allograft (11).

Febuxostat-associated renal injury and PH2-related oxalate nephropathy are distinct disease processes that may coexist and share overlapping clinical and pathological features, thereby complicating diagnosis. Febuxostat lowers UA and may alter urinary solute composition, potentially triggering crystal deposition in a kidney burdened by oxalate overproduction. In such cases, the initial presentation can mimic febuxostat-associated acute tubular injury, leading clinicians to anchor on a drug-related diagnosis and overlook an underlying metabolic disorder.

Here we report a case in which febuxostat, used for post-transplant hyperuricemia, appears to have acted as a metabolic trigger that unmasked underlying PH2, leading to recurrent oxalate nephropathy in the renal allograft. This case highlights a diagnostic trap and underscores the importance of early metabolic screening and genetic testing in kidney transplant recipients with unexplained allograft dysfunction and crystalline nephropathy.

## 2 Case report

### 2.1 Patient history and febuxostat initiation

A 67-year-old woman with end-stage renal disease was admitted for kidney transplantation. She was initially diagnosed with sepsis, nephrolithiasis, and renal insufficiency approximately three years before transplantation, with a serum creatinine (Scr) of approximately 600 μmol/L. After anti-infective treatment and bilateral nephrolithotomy, she initiated hemodialysis and remained on regular hemodialysis until transplantation. The transplant surgery procedure was smooth. Post-transplant immunosuppression consisted of tacrolimus (dynamically adjusted to a target concentration of 8–12 ng/mL, as indicated in **Supplementary Figure S1**), mycophenolate mofetil (MMF), corticosteroids, and rabbit anti-human thymocyte immunoglobulin. Renal function recovered well, with Scr declining from 524 μmol/L to a nadir of 116.4 μmol/L by approximately one month after transplantation. However, postoperative serum UA levels progressively increased two months after transplantation, peaking at approximately 519 μmol/L. For management of persistent post-transplant hyperuricemia, the patient was started on febuxostat 40 mg once daily.

### 2.2 Progressive allograft dysfunction and diagnostic evaluation

**Figure 1** shows the clinical course and laboratory trends, and **Table 1** summarizes the timeline of key laboratory parameters and clinical events after kidney transplantation. The UA level decreased to 206.5 μmol/L after 9 days post- febuxostat initiation. Despite outpatient monitoring and empirical tacrolimus dose adjustments, creatinine continued rising, reaching 242.0 μmol/L approximately 5 months after starting febuxostat. The patient was readmitted with a Scr of 261 μmol/L. Physical examination revealed extremity edema but no fever, jaundice, petechiae, ecchymoses, superficial lymphadenopathy, allograft swelling, or tenderness.

**Figure 1:**
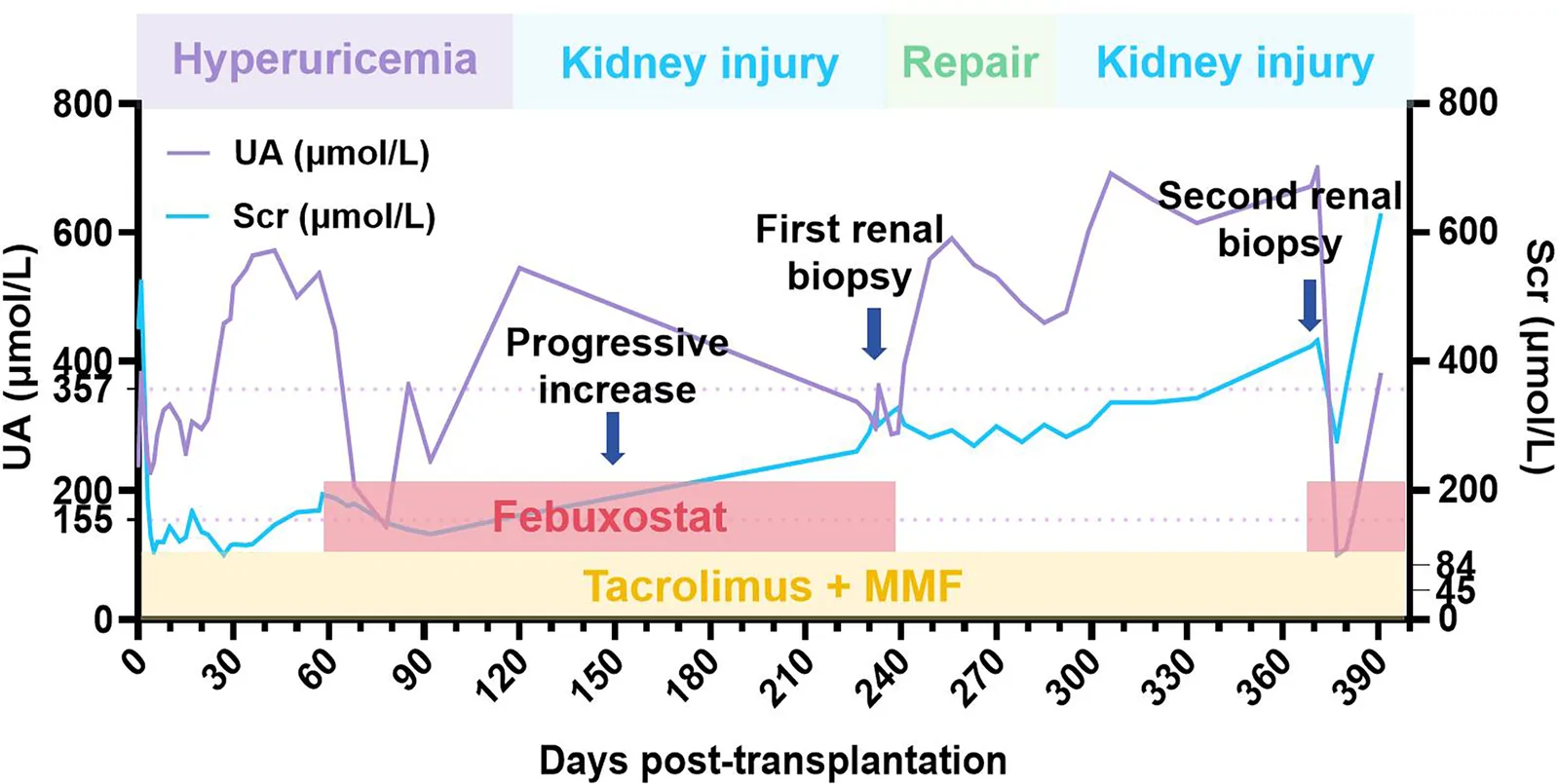
Variation of UA and Scr levels with annotated clinical events post-transplantation.

**Table 1:** Timeline of key laboratory parameters and clinical events after kidney transplantation.

Days post-transplantation	Scr (μmol/L)	eGFR (mL/min/1.73m²)	UA (μmol/L)	Tacrolimus (ng/mL)	Albumin (g/L)	Calcium (mmol/L)	Clinical event
-1	450.2	8.17	236.1	—	29.7	1.74	Dialysis-dependent, admitted for transplantation
0	524	6.8	382.1	—	28.4	1.76	Transplantation
30	116.4	41.9	516.4	15.4	36.2	1.61	Gradual recovery of renal graft function post-transplantation
58	194.0	22.59	519.0	—	28.4	1.68	Second admission (edema, fatigue); hyperuricemia
60	—	—	—	—	—	—	Febuxostat initiated at 40 mg/d
68	179.6	24.8	206.5	4.4	36.7	1.95	9 days post-febuxostat; Scr rising
226	261.0	15.8	338.0	5.1	31.2	2.04	Third admission
230	290.1	13.89	318.9	4.0	31.7	1.90	First renal biopsy
238	—	—	—	—	—	—	Febuxostat discontinued
239	327.9	11.98	289.8	7.2	36.2	2.12	Scr increased, ~2.8 times the baseline
278	275.5	—	489	4.69	31.3	1.83	40 days post-discontinuation; Scr decreased
369	424	8.72	672	10.0	31.3	1.78	Fourth admission
371	433	8.5	700	6.6	32.9	1.50	Second renal biopsy
373	—	—	—	—	—	—	Hemodialysis initiated; febuxostat restarted at 20 mg QD
377	277	14.59	100	6.1	31.5	1.93	Post-dialysis improvement
378	—	—	—	—	—	—	24h urinary oxalate 65.74 mg/24h; genetic testing confirmed PH2
391	630.2	5.40	382.5	—	34.4	1.84	Fifth admission

Reference ranges: Scr 45–84 μmol/L; UA 155–357 μmol/L; albumin 40–55 g/L; calcium 2.11–2.52 mmol/L. “—” indicates data not available.

A comprehensive diagnostic work-up was undertaken to identify the cause of progressive allograft dysfunction. Common etiologies were systematically excluded. Donor-specific antibodies and panel-reactive antibodies were negative, excluding antibody-mediated rejection. BK virus DNA in urine was elevated (1.30×10^8^ copies/mL), but BK virus DNA in plasma was negative, and SV40 immunostaining in renal biopsy was negative, excluding BK virus-induced nephropathy. As shown in **Supplementary Figure S1**, 3 months post-transplantation, plasma tacrolimus concentrations ranged from 4.0 to 8.2 ng/mL, entirely within the therapeutic target range (4–10 ng/mL). Electron microscopy showed no characteristic uniformly sized vacuolization, excluding calcineurin inhibitor toxicity. PCR testing for cytomegalovirus (CMV) and Epstein-Barr virus (EBV) from blood and urine samples was negative. Renal Doppler ultrasound examination showed no hydronephrosis or perigraft collection, although arterial resistive indices were elevated (0.74–0.86). Despite supportive care, Scr continued to rise, peaking at 327.9 μmol/L about 5.5 months after starting febuxostat.

### 2.3 First renal allograft biopsy and initial management

A percutaneous renal allograft biopsy was performed when the Scr rise could not be explained by the above causes. **Supplementary Table S1** lists the biopsy findings of the transplant kidney. The most striking findings involved the tubulointerstitium: acute tubular injury with epithelial swelling, nuclear loss, brush border loss, vacuolization, and focal necrosis. Tubular atrophy involved ~25% of the tubular area (ct1), with no tubulitis (t0). Interstitial fibrosis occupied ~10% of the biopsy area (ci1), with mild mononuclear cell infiltration (i1) and no granulomas or eosinophil predominance. Immunofluorescence showed no significant immune complex deposition, and C4d staining was negative. Immunohistochemistry showed a macrophage-predominant infiltrate (CD68^+^, 10% in focal areas), with only scattered T cells (CD3^+^, 5%); SV40 was negative, excluding BK virus nephropathy. Electron microscopy revealed no electron-dense deposits but did show peritubular capillary basement membrane multilayering (2–3 layers) in 5 of 6 capillaries examined. Collectively, the final pathological diagnosis was as follows: no definite T cell-mediated rejection; no definite BK virus nephropathy; acute tubular injury with tubular calcification and crystal formation, suspected to be crystalline drug or phosphate/oxalate deposition. As shown in **Figures 2A–C**, crystal formation was identified within the tubular lumen.

**Figure 2:**
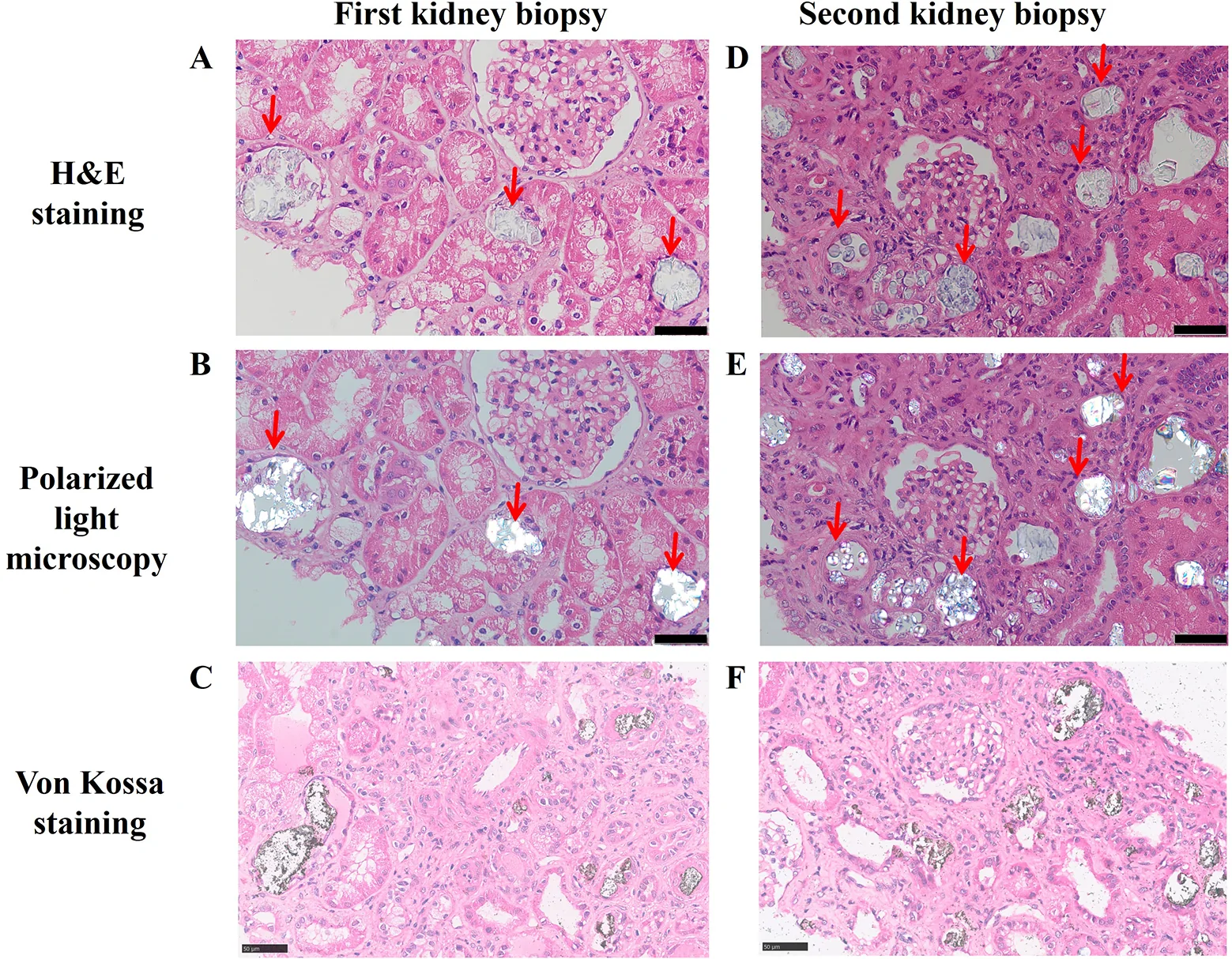
Histopathological findings of two renal allograft biopsies. **(A–C)** First biopsy (post-transplant day 230): **(A)** H&E staining observed with light microscopy showing acute tubular injury with crystalline material (arrows); **(B)** Polarized light microscopy showing birefringence; **(C)** Von Kossa staining positive for calcium deposition; **(D–F)** Second biopsy (post-transplant day 371): **(D)** H&E staining showing abundant intratubular crystals (arrows); **(E)** Polarized light microscopy suggesting polychromatic birefringence characteristic of calcium oxalate; **(F)** Von Kossa staining strongly positive; Scale bars = 50 μm. Original magnification: ×400 for **(A–F)**.

The clinical pharmacist, in collaboration with the treatment team, conducted a comprehensive medication review for this patient (**Supplementary Table S2**), which was suggestive of an adverse drug reaction. Febuxostat was the only medication initiated within the temporal window preceding the progressive rise in Scr; no other nephrotoxic agents (non-steroidal anti-inflammatory drugs, contrast media, aminoglycosides) had been administered. Febuxostat was discontinued. Following withdrawal, the progressive decline in renal function was arrested: Scr decreased from 327.9 μmol/L to 275.5 μmol/L after 40 days (**Figure 1**, **Table 1**). Although the UA level rebounded after discontinuing febuxostat, it eventually trended downward. The patient was satisfied with the treatment outcome and was discharged.

At follow-up, Scr re-elevated to 371.5 μmol/L, with tacrolimus concentration of 9.09 ng/mL, albumin 25.8 g/L, and UA 689 μmol/L; she was readmitted and underwent a second renal allograft biopsy to further clarify the cause of the disease.

### 2.4 Second renal allograft biopsy and metabolic evaluation

Compared with the first biopsy, the second renal allograft biopsy showed progression of tubular atrophy (from approximately 25% to 50% of the tubular area; ct1 to ct2). Under polarized light microscopy, abundant crystalline material was observed within the renal tubules, and the crystals were suspected to be compatible with calcium oxalate (**Figures 2D, E**). Von Kossa staining was positive in both biopsy specimens, suggesting a mixed calcium oxalate/calcium phosphate composition rather than pure calcium oxalate (**Figures 2C, F**). Metabolic evaluation (24-h urine) revealed markedly elevated urinary oxalate excretion (65.74 mg/24h; reference: < 40 mg/24h), with low urinary citrate (0.25 mg/24h) and low urinary calcium (0.08 mmol/d).

### 2.5 Genetic diagnosis, treatment, and outcome

Genetic testing identified a rare likely pathogenic variant in the GRHPR gene, consistent with a diagnosis of PH2. Genetic testing used next-generation sequencing targeting genes associated with inherited kidney stone disorders and PH. The analysis identified a homozygous splice-site variant, c.599-1G>C (NM_012203.2), in the GRHPR gene. ClinVar (VCV000555266.3) lists this variant as likely pathogenic and associates it with primary hyperoxaluria type II. The biallelic homozygous genotype, together with the genotype-phenotype correlation, supports the pathogenic role of this rare variant in this PH2 case. The mixed crystalline composition further supports PH2 with secondary calcium phosphate deposition. To our knowledge, no previous report was identified describing GRHPR variant c.599-1G>C in a kidney transplant recipient presenting with crystalline nephropathy. To support the novelty of this case, we performed a systematic literature search in PubMed, Embase, Web of Science, CNKI, and Wanfang Data from inception to September 2026. Search terms included “primary hyperoxaluria”, “primary hyperoxaluria type II”, “PH2”, “GRHPR”, and “kidney transplantation”. Reference lists of retrieved articles were also screened.

The patient was started on potassium citrate and vitamin B6. Despite supportive care, renal function continued to decline (Scr 630.2 μmol/L). On day 373 post-transplantation, she initiated hemodialysis and restarted febuxostat at 20 mg once daily because of persistent hyperuricemia. The rechallenge was continued for approximately 4 weeks. Serial urine pH measurements are shown in **Supplementary Figure S2**. Before febuxostat initiation, urine pH was 5.5–6.0. During febuxostat therapy, it was observed that urine pH increased to 8.0 at approximately post-transplant day 90, then gradually declined to 5.0 by day 240. After febuxostat was restarted on post-transplant day 373, urine pH increased again to 7.5. The patient then established an arteriovenous fistula in the nephrology department and was discharged from the hospital on day 406 post-transplantation. **Table 2** summarizes the differential diagnosis of progressive allograft dysfunction.

**Table 2:** Differential diagnosis of progressive allograft dysfunction.

Diagnosis	Diagnostic test performed	Result	Conclusion
T cell-mediated rejection	Renal biopsy (Banff 2019)	i1, t0, g0, v0; no tubulitis	Excluded
Antibody-mediated rejection	Donor-specific antibodies	Negative; C4d negative	Excluded
BK virus nephropathy	Urine BK DNA; Biopsy SV40	Urine BK 1.30E8 cp/mL; SV40 negative	Excluded (viral shedding only)
Calcineurin inhibitor toxicity	Tacrolimus levels; Biopsy EM	Levels 4.0–8.2 ng/mL; No EM isometric vacuolization	Excluded
Infection	Blood/urine culture; CMV/EBV PCR	Negative	Excluded
Recurrent/*de novo* GN	Immunofluorescence	Negative for immune complexes (see **Supplementary Table S1** for details)	Excluded
Obstruction	Renal ultrasound	No hydronephrosis	Excluded
Drug-induced tubular injury	Medication reconciliation; Biopsy pathology	Initial suspicion (febuxostat is temporally associated; Biopsy indicated acute tubular injury, with crystalline deposition suspected); crystals persisted after drug withdrawal	Not the primary etiology
PH2	Urinary oxalate, genetic testing	Urinary oxalate 65.74 mg/24h; GRHPR variant	Probable

BK, BK polyomavirus; EM, electron microscopy; GN, glomerulonephritis.

## 3 Discussion

### 3.1 Adverse drug reactions related to febuxostat: comparison with published literature

Previous studies have described febuxostat-associated kidney injury primarily in non-transplant populations (4). The most frequently documented adverse reactions include liver dysfunction, hyperlipidemia, and diarrhoea during clinical trials (2, 12–16). A brief literature review reveals important differences between published cases and our patient (**Supplementary Table S3**). Notably, our patient lacked fever, rash, or peripheral eosinophilia, features typically associated with drug-induced interstitial nephritis. Instead, the biopsy demonstrated predominant acute tubular injury with a macrophage-predominant infiltrate (CD68^+^ 10%), a pattern more consistent with direct tubular toxicity or crystalline nephropathy than classic hypersensitivity-mediated injury.

The pathological evidence of suspected crystalline drug or metabolite deposition is notable. Polarization microscopy identified crystals that may represent drug or phosphate/oxalate deposition rather than urate. Febuxostat and its metabolites have low aqueous solubility, and intratubular crystal formation is a proposed mechanism of nephrotoxicity in preclinical models. Persistent hypocalcemia (1.61–1.77 mmol/L) during febuxostat therapy may have promoted calcium-phosphate crystallization in the alkaline urine induced by its uricosuric effect. Several factors may have increased susceptibility to drug-associated tubular injury: i) Ischemia-reperfusion injury and persistently elevated renal arterial resistive indices (peak 0.86) suggest microvascular dysfunction that may amplify febuxostat toxicity. ii) Chronic hypoalbuminemia (as low as 26.6 g/L) may increase the free fraction of highly protein-bound febuxostat (~99%), enhancing tubular exposure (17, 18). iii) Febuxostat inhibits xanthine oxidase and increases urinary hypoxanthine and xanthine, which have limited solubility in acidic urine but may precipitate in alkaline urine.

The Naranjo Adverse Drug Reaction Probability Scale was applied (**Supplementary Table S4**). The score was 4 points (“possible”) based on temporal association, improvement after withdrawal, and previous reports of febuxostat-associated kidney injury. After the diagnosis of PH2 was established, the causal role of febuxostat was re-evaluated. The reintroduction of febuxostat after dialysis initiation did not allow a valid assessment of drug causality, because the rechallenge occurred after substantial allograft dysfunction had already developed. Therefore, the Naranjo item for rechallenge remained scored as 0 (“not assessed”). This further weakens any causal inference between febuxostat and the original renal injury. The score decreased from the initial 7 (probable) to 4 (possible), reflecting that PH2 is an independent and sufficient cause of oxalate nephropathy. Febuxostat may have acted as a metabolic trigger rather than the primary etiologic agent.

### 3.2 Diagnostic trap: from drug toxicity to genetic disease

The initial clinical course strongly suggested febuxostat-associated acute tubular injury. The temporal association between drug initiation and progressive renal dysfunction, the presence of crystalline material on the first biopsy, and the temporary stabilization after drug withdrawal all pointed to drug toxicity as the primary etiology. However, the subsequent re-elevation of Scr despite febuxostat discontinuation, the marked rebound of UA, and the abundant calcium oxalate crystals on the second biopsy suggested an underlying metabolic rather than drug-induced pathology. This case illustrates a common diagnostic trap: when a plausible drug-related explanation exists, clinicians may anchor on this diagnosis and fail to pursue underlying metabolic disorders. The persistence of crystals on the second biopsy at four months after febuxostat withdrawal supports that the oxalate overproduction was related to a metabolic factor.

### 3.3 The role of febuxostat: trigger or bystander?

The precise role of febuxostat in this case warrants careful reconsideration. Febuxostat has not been proven to exacerbate hyperoxaluria. In fact, xanthine oxidase inhibitors have been shown to reduce calcium oxalate stone recurrence in patients with hyperuricosuria. In our patient, febuxostat effectively controlled serum UA but did not prevent progressive renal dysfunction. In this patient, urine pH increased to 8.0 during febuxostat therapy and later rose again after febuxostat was restarted (**Supplementary Figure S2**). These observations are compatible with a possible effect of febuxostat on urinary pH. We further hypothesize that an alkaline urinary environment could have promoted calcium phosphate precipitation, which might then have served as a nidus for calcium oxalate deposition. However, urinary solute composition and crystal nucleation were not directly measured, and this mechanism remains speculative. This “second-hit” model is consistent with the temporary stabilization after drug withdrawal, followed by re-elevation as the underlying metabolic defect continued unabated. The established diagnosis of PH2 is supported by genetic confirmation and persistent crystalline deposition. The contributory role of febuxostat remains a hypothesis.

### 3.4 Diagnostic value of Von Kossa staining and polarized light microscopy

Multiple modalities supported mixed calcium oxalate/calcium phosphate crystals. Under polarized light microscopy, the crystals exhibited bright birefringence with characteristic polychromatic colors, compatible with calcium oxalate. However, Von Kossa staining was positive in both biopsy specimens, indicating the possible presence of calcium phosphate. This combination suggests a mixed crystal composition rather than pure calcium oxalate. Together with elevated urinary oxalate excretion, this combination provides findings compatible with mixed crystalline nephropathy in the setting of PH2. In future cases, when oxalate nephropathy is suspected, we recommend measurement of 24-h urinary oxalate. Genetic testing for PH should be considered in patients with unexplained oxalate nephropathy, particularly those with a history of nephrolithiasis.

### 3.5 Clinical implications for transplant practice

The diagnosis of PH2 has important therapeutic implications. For patients with PH2 and end-stage renal disease, combined liver-kidney transplantation offers a potential cure by correcting the underlying hepatic metabolic defect (11). Isolated kidney transplantation, by contrast, carries a high risk of recurrent oxalate nephropathy and graft failure (7). In our patient, the diagnosis of PH2 was established after the allograft had already failed, highlighting the importance of early recognition. We recommend that clinicians maintain a high index of suspicion for PH in kidney transplant recipients with unexplained allograft dysfunction and crystalline nephropathy, particularly those with a history of nephrolithiasis of unclear etiology. Early metabolic screening and genetic testing can prevent diagnostic delay and guide timely consideration of combined liver-kidney transplantation.

### 3.6 Limitations

The exact mechanism by which febuxostat may have contributed to crystal deposition in this patient with PH2 remains speculative. Stone composition analysis from the patient’s prior nephrolithiasis was not available. Additionally, FTIR or Raman spectroscopy was not performed on the biopsy specimens, and the exact proportion of calcium oxalate versus calcium phosphate in the mixed crystals could not be quantified. Urinary pH was measured intermittently, and solute composition was not directly assessed. Therefore, the proposed mechanism linking febuxostat, urinary pH changes, and crystal formation remains hypothetical. However, polarized light microscopy, Von Kossa staining, and clinical/metabolic correlation provide certain evidence for mixed calcium oxalate/calcium phosphate crystalline nephropathy.

## 4 Conclusion

This case demonstrates that PH2 can present as recurrent oxalate nephropathy after isolated kidney transplantation. Febuxostat may have contributed to the clinical presentation, but the established diagnosis is PH2, and the causal role of febuxostat is not proven. The presence of mixed calcium oxalate/calcium phosphate crystals suggests that calcium phosphate may serve as a nidus for calcium oxalate deposition. The increase in urinary pH observed during febuxostat therapy may have contributed to calcium phosphate precipitation, although this remains speculative. Genetic testing established the diagnosis by revealing a rare pathogenic GRHPR variant. This case highlights a diagnostic trap: the initial presentation mimicked febuxostat-associated acute tubular injury, but the subsequent clinical course and genetic testing revealed PH2 as the true underlying etiology. The second biopsy showed progression of tubular atrophy and interstitial fibrosis, indicating that a substantial portion of the injury had already become irreversible. The final renal outcome was end-stage allograft failure requiring permanent hemodialysis. This incomplete recovery underscores the importance of early recognition and intervention. Clinicians should consider PH in kidney transplant recipients with unexplained allograft dysfunction and calcium oxalate crystal deposition on biopsy, particularly when renal function fails to recover after drug withdrawal or when UA rebounds markedly. Early metabolic screening and genetic confirmation are essential to avoid diagnostic delay and to guide appropriate management, including consideration of combined liver-kidney transplantation.

## Patient perspective

The patient experienced physical decline and extremity edema during the period of unexplained renal function decline despite immunosuppressive therapy. Following the diagnosis of PH2 and adjustment of treatment, including initiation of hemodialysis and metabolic therapy, her symptoms gradually improved. The patient expressed satisfaction with the treatment outcome and shared her clinical course to improve understanding of this challenging condition.

## Data Availability

The original contributions presented in the study are included in the article/**Supplementary Material**. Further inquiries can be directed to the corresponding author.
