# Supplementary material for: Primary hyperoxaluria type 2 presenting with febuxostat-associated acute tubular injury in a kidney transplant recipient: a case report
Source: Front Immunol. 2026 Oct 28;17:1932592. doi: 10.3389/fimmu.2026.1932592 (PMC13751349; doi:10.3389/fimmu.2026.1932592)
Supplement: Supplementary file 1 [file Table1.docx]

# **Supporting materials**

**Supporting figures and tables**

Figure S1. The concentration of tacrolimus post-transplantation.

Figure S2. The changes in urinary pH over time post-transplantation.

Table S1. Summary of transplant kidney biopsy findings.

Table S2. Summary of major medications.

Table S3. Comparison with published literature.

Table S4. Naranjo score of febuxostat and the occurrence of kidney injury as an adverse reaction.

### **Figure S1. The concentration of tacrolimus post-transplantation.** The purple area represents the suggested concentration range.

### **Figure S2. The changes in urinary pH over time post-transplantation.** Urine pH fluctuated during febuxostat therapy, rising to 8.0 at approximately post-transplant day 90 and declining thereafter. After febuxostat was restarted on post-transplant day 373, urine pH rose again to 7.5. Shaded areas indicate periods of febuxostat administration.

### **Table S1. Summary of transplant kidney biopsy findings**

| **Category** | **Parameter/Lesion** | **Description** | **Banff 2019 Score** |
| --- | --- | --- | --- |
| Glomeruli | Global sclerosis | None | – |
|  | Endocapillary infiltration | None | g0 |
|  | Double contours of GBM | None | cg0 |
|  | Mesangial matrix expansion | Mild | mm0-1 |
| Arteries / Arterioles | Hyaline arteriolosclerosis | None | ah0 |
|  | Arterial intimal fibrosis | None | cv0 |
|  | Endarteritis | None | v0 |
|  | Microthrombi | None | – |
| Tubules | Acute tubular injury | Epithelial swelling, nuclear loss, loss of brush border, calcification, crystal deposition | – |
|  | Tubular atrophy | Approximately 25% | t0, ct1 |
|  | Lymphocytic infiltration within the tubular epithelium | None | – |
|  | BK virus-like inclusions | None (SV40^–^) | – |
| Interstitium | Fibrosis | Approximately 10% | ci1 |
|  | Inflammatory cell infiltration | Mild | i1 |
| Peritubular capillaries | Mononuclear cells | None | ptc0 |
|  | C4d deposition | Negative | C4d– |
| Immunofluorescence | IgG | Positive | – |
|  | IgA | Positive | – |
|  | IgM, C3c, C4c, C1q | Negative | – |
|  | Fibrinogen | Positive | – |
|  | κ, λ | Negative | – |
| Immunohistochemistry | CD3, CD4, CD8, CD79α | Dot-like positive, ~5% | – |
|  | CD68 | Focally positive, ~10% | – |
|  | HLA‑DR | Dot-like and linear positive, ~10% | – |
|  | SV40 | Negative | – |
| Electron microscopy | GBM thickness | Diffuse thickening (459–653 nm), no electron‑dense deposits | – |
|  | Podocytes | Vacuolization, focal foot process effacement | – |
|  | Mesangium | Mild segmental expansion, no electron‑dense deposits | – |
|  | PTC basement membrane layering | 5/6 capillaries with 2‑3 layers | – |

Abbreviations: “–”, not scored/not applicable; Banff 2019, Banff Classification 2019; GBM, glomerular basement membrane; g0, no glomerulitis; cg0, no chronic glomerulopathy; mm0–1, mild mesangial matrix expansion; ah0, no hyaline arteriolosclerosis; cv0, no arterial intimal fibrosis; v0, no endarteritis; t0, no tubulitis; ct1, tubular atrophy 1; ci1, interstitial fibrosis 1; i1, interstitial inflammation 1; ptc0, no peritubular capillaritis; C4d, complement fragment C4d; SV40, simian virus 40 large T antigen; Ig, immunoglobulin; IgG/IgA/IgM, immunoglobulin G/A/M; C3c/C4c/C1q, complement components/fragments; κ/λ, kappa/lambda light chains; CD, cluster of differentiation; CD3, pan-T-cell marker; CD4, helper T-cell marker; CD8, cytotoxic T-cell marker; CD79α, B-cell marker; CD68, macrophage/histiocyte marker; HLA DR, human leukocyte antigen DR; PTC, peritubular capillary; BK virus, BK polyomavirus.

### **Table S2. Summary of major medications**

| **Hospitalization** | **Medications (drug, time post-transplant, dose)** |
| --- | --- |
| Hospitalization #1 (POD 0–30) | Tacrolimus, POD 0–30, 3→2→1 mg, BIDAC/ QD(06) + QN/ BID;  MMF, POD 0–30, 0.75–1 g, BIDAC/ QD(06) + QN;  Prednisone, POD 7–30, 20 mg QD(06);  Methylprednisolone, POD 0–7, 500→40 mg tapering;  Rabbit anti-human thymocyte immunoglobulin, POD 0–22, 5–10 mg multiple doses;  IVIG, POD 0–8, 200 mL QD;  Human albumin, POD 0–8, 100 mL BID→QD;  Piperacillin/tazobactam, POD 0–20, 4.5 g Q12H;  Valacyclovir, POD 13–28, 300 mg BID;  Ganciclovir, POD 28–30, 0.5 g BID;  Levofloxacin, POD 30, 0.5 g QD single;  Trimethoprim/sulfamethoxazole, POD 30, 1 co BID;  Furosemide, POD 0–30, 20 mg BID intermittent;  Torsemide, POD 0–14, 20 mg QD; POD 14, 10 mg;  Tolvaptan, POD 7–30, 7.5→15 mg QD;  Calcium gluconate, POD 1–26, 2 g QD multiple;  Sodium bicarbonate tablets, POD 16–30, 1 g TID;  Sodium bicarbonate injection, POD 12, 14, 25, 125–250 mL;  Calcium carbonate/vitamin D3, POD 15–30, 600 mg BID;  Calcitriol, POD 30, 0.25 μg QD;  Potassium chloride, POD 2, 7, 9, 3 g;  Erythropoietin, POD 21–30, 10,000 IU TIW1;  Enarodustat, POD 21–30, 6 mg QD;  Wuzhi capsules, POD 26–30, 11.25 mg BID;  Esomeprazole, POD 0–11, 40 mg BID;  Omeprazole, POD 11–30, 20 mg QD;  Lactulose, POD 0–21, 10–30 mL QD;  Saccharomyces boulardii, POD 11–30, 500 mg BID;  Papaverine, POD 0–19, 60 mg Q12H;  Alprostadil, POD 0–19, 10 μg QD;  Metaraminol, POD 7–14, 50 mg QD;  Norepinephrine, POD 2–7, 6 mg;  Insulin, POD 0–4, 8 IU Q12H/QD;  Nalbuphine, POD 0–1, 20 mg;  Dexmedetomidine, POD 0–1, 0.4 mg |
| Hospitalization #2 (POD 56–69) | Tacrolimus, POD 56, 1 mg BID;  MMF, POD 56, 1 g BID;  Prednisone, POD 56, 5 mg QD(06);  Mycophenolate sodium, POD 69, 180 mg BID;  Torsemide, POD 56–69, 10 mg QD;  Spironolactone, POD 66–69, 20 mg BID;  Human albumin, POD 56–69, 100 mL QD;  Calcium gluconate, POD 61–69, 2 g QD;  Sodium bicarbonate tablets, POD 59, 0.5 g TID;  Febuxostat, POD 59–69, 40 mg QD;  Potassium sodium hydrogen citrate, POD 60–69, 2.5g BID + 5g QN;  Enarodustat, POD 68–69, 6 mg QD;  Pegylated G-CSF, POD 68, 3 mg;  Omeprazole, POD 56–69, 40 mg QD;  Oryz-Aspergillus enzyme and pancreatin tablets, POD 59–69, 1 tablet TID;  Whole protein enteral nutrition, POD 63, 50 g BID;  Rivaroxaban, POD 69, 10 mg QD |
| Hospitalization #3 (POD 225–240) | Tacrolimus, POD 225–240, 1–2 mg, QD(06) + QN/BIDAC;  MMF, POD 225–240, 0.25 g BID;  Prednisone, POD 225–240, 5 mg QD(06);  Methylprednisolone, POD 234, 300 mg;  IVIG, POD 230–236, 100–200 mL QD;  Human albumin, POD 227–236, 100 mL QD;  Furosemide, POD 225–237, 20 mg QD, oral then IV;  Sodium bicarbonate tablets, POD 236–240, 1 g TID;  Sodium bicarbonate injection, POD 236, 238, 239, 200 mL;  Febuxostat, POD 225–237, 40 mg QD;  Diyu Sheng Bai tablets, POD 227–240, 200–400 mg TID;   G-CSF, POD 231, 236, 238, 200 μg;   Esomeprazole, POD 234–236, 40 mg BID |
| Hospitalization #4 (POD 366–384) | Tacrolimus, POD 366–384, 1–2.5 mg, Q12H/ QD(06) + QN;  MMF, POD 366–384, 0.25 g BID;  Prednisone, POD 366–384, 5 mg QD(06);  Torsemide, POD 372–381, 10 mg QD;  Tolvaptan, POD 381–384, 15 mg QD;  Human albumin, POD 367–384, 100 mL QD;  IVIG, POD 384, 100 mL;  Calcium gluconate, POD 372–384, 2 g QD;  Sodium bicarbonate tablets, POD 366–373, 1 g TID;  Sodium bicarbonate injection, POD 369, 370, 371, 250 mL;  Febuxostat, POD 372–384, 20 mg QD;  Calcitriol, POD 373–384, 0.25 μg QD;  Potassium sodium hydrogen citrate, POD 373–384, 2.5g TID;  Vitamin B6, POD 373–384, 10 mg QD;  Roxadustat, POD 384, 50 mg TIW1;  Diyu Sheng Bai tablets, POD 366–384, 400 mg TID;  Whole protein enteral nutrition, POD 374, 50 g TID |
| Hospitalization #5 (POD 390–399) | Tacrolimus, POD 390–406, 1 mg, BID;  MMF, POD 390–406, 0.25 g BID;  Prednisone, POD 390–406, 5 mg QD(06);  Febuxostat, POD 390–406, 20 mg QD;  Vitamin B6, POD 390–406, 10 mg QD;  Calcitriol, POD 373–384, 0.25 μg QD;  Piperacillin/tazobactam, POD 391–399, 4.5 g BID;  Budesonide, POD 391–397, 2 ml BID nebulized;  Acetylcysteine, POD 391–397, 0.3 g BID nebulized;  Eucalyptol/limonene/pinene enteric capsules, POD 393–399, 0.3 g TID;  Vonoprazan, POD 397–399, 20 mg QD;  CSF, POD 393, 100 μg;  Fat emulsion/amino acids/glucose, POD 399, 1000 ml QD |

**Note:** Time is expressed as post-transplant days (POD). Doses are the main recorded ranges. Abbreviations: QD = once daily, BID = twice daily, TID = three times daily, Q12H = every 12 hours, QN = every night, QD(06) = at 06:00, BIDAC = twice daily before meals, TIW1 = once weekly.

### **Table S3. Comparison with published literature**

| **Characteristic** | **Published cases** | **Our case** |
| --- | --- | --- |
| Population | CKD or gout patients | Kidney transplant recipient |
| Pathological diagnosis | Clinical diagnosis or acute interstitial nephritis | Acute tubular injury |
| Hypersensitivity features (rash, fever, eosinophilia) | Case reported (13, 14, 16) | Absent |
| Time to onset after febuxostat initiation | Days to weeks | Months (insidious onset) |
| Response to drug withdrawal | Usually complete recovery | After a brief period of stability, it continued to rise |
| Risk factors identified | Renal impairment, allopurinol hypersensitivity (15) | Hypoalbuminemia (chronic), elevated vascular resistance indices |

### **Table S4. Naranjo score of febuxostat and the occurrence of kidney injury adverse reaction**

| **No.** | **Items** | **Yes** | **No** | **Unknown or not examined** | **Naranjo score** |
| --- | --- | --- | --- | --- | --- |
| 1 | Are there clear reports that have previously reported this effect? | +1 | 0 | 0 | +1 |
| 2 | Has the adverse event developed after administration of the suspected drug? | +2 | −1 | 0 | +2 |
| 3 | Has the adverse effect begun to improve when medication is released or a special antidote is applied? | +1 | 0 | 0 | +1 |
| 4 | Did the adverse effect repeat when the drug was applied again? | +2 | −1 | 0 | 0 |
| 5 | Are there alternative reasons that may cause impact? | −1 | +2 | 0 | −1 |
| 6 | Did the effect reappear when placebo was given? | −1 | +1 | 0 | 0 |
| 7 | Is the drug detected at toxic concentrations in any body fluid? | +1 | 0 | 0 | 0 |
| 8 | Has the effect increased when the dose is increased, or is the effect decreased when it is decreased? | +1 | 0 | 0 | 0 |
| 9 | Is there a similar history of effect against a same or similar medication in patient resume? | +1 | 0 | 0 | 0 |
| 10 | Has the adverse event been evidenced by any objective evidence? | +1 | 0 | 0 | +1 |
| **Score** |  |  |  |  | **4** |

Note: >9: Definite adverse event; 5–8: Probable adverse event; 1–4: Possible adverse event; 0: Suspicious adverse event.
